# High Seroprevalence of Human Herpesviruses in HIV-Infected Individuals Attending Primary Healthcare Facilities in Rural South Africa

**DOI:** 10.1371/journal.pone.0099243

**Published:** 2014-06-10

**Authors:** Erik Schaftenaar, Georges M. G. M. Verjans, Sarah Getu, James A. McIntyre, Helen E. Struthers, Albert D. M. E. Osterhaus, Remco P. H. Peters

**Affiliations:** 1 Department of Viroscience, Erasmus Medical Center, Rotterdam, The Netherlands; 2 Anova Health Institute, Johannesburg and Tzaneen, South Africa; 3 School of Public Health & Family Medicine, University of Cape Town, Cape Town, South Africa; 4 Department of Medicine, Division of Infectious Diseases & HIV Medicine, University of Cape Town, Cape Town, South Africa; 5 Department of Medical Microbiology, Maastricht University Medical Centre, Maastricht, The Netherlands; University of British Columbia, Canada

## Abstract

Seroprevalence data of human herpesviruses (HHVs) are limited for sub-Saharan Africa. These are important to provide an indication of potential burden of HHV-related disease, in particular in human immunodeficiency virus (HIV)-infected individuals who are known to be at increased risk of these conditions in the Western world. In this cross-sectional study among 405 HIV-infected and antiretroviral therapy naïve individuals in rural South Africa the seroprevalence of HHVs was: herpes simplex virus type 1 (HSV-1) (98%), herpes simplex virus type 2 (HSV-2) (87%), varicella zoster virus (VZV) (89%), and 100% for both Epstein-Barr virus (EBV) and cytomegalovirus (CMV). Independent factors associated with VZV seropositivity were low educational status and having children. Lack of in-house access to drinking water was independently associated with positive HSV-1 serostatus, whereas Shangaan ethnicity was associated with HSV-2 seropositivity. Increasing age was associated with higher IgG titres to both EBV and CMV, whereas CD4 cell count was negatively associated with EBV and CMV IgG titres. Moreover, IgG titres of HSV-1 and 2, VZV and CMV, and CMV and EBV were positively correlated. The high HHV seroprevalence emphasises the importance of awareness of these viral infections in HIV-infected individuals in South Africa.

## Introduction

Herpes simplex virus type 1 (HSV-1) and 2 (HSV-2), varicella zoster virus (VZV), Epstein-Barr virus (EBV), and cytomegalovirus (CMV) are human herpesviruses (HHV) that are prevalent worldwide. HHV Infections can lead to a variety of clinical conditions that range in severity from cold sores and genital ulcers (HSV), and chickenpox (VZV) to potentially sight-threatening (e.g. CMV uveitis and HSV keratitis) or even life-threatening diseases such as HSV encephalitis and EBV-associated malignancies [1]–[3]. Primary HHV infections, commonly acquired at young age, lead to a life-long latent infection with intermittent reactivation resulting in periodic asymptomatic or recrudescent disease. The host immune system is pivotal to resolve lytic infections and to inhibit HHV reactivation from latency. Consequently, reactivation of HHV is much more frequent among immunocompromised individuals including those infected with human immunodeficiency virus (HIV) [4], [5]. Also, HIV-infected individuals have an increased risk of developing more severe HHV-related disease [4], [5]. Even after the introduction of antiretroviral therapy (ART), HIV-infected individuals remain at risk of developing severe HHV-related diseases as ART-induced recuperation of adaptive immunity may result in vigorous anti-HHV cellular immune responses. In the case of residual ocular CMV infection, restored CMV immunity may lead to sight-threatening immune recovery uveitis [6]. HHV infections clinically interact with HIV, contribute substantially to hospitalization, morbidity and mortality and some HHVs (e.g. HSV-2) may even facilitate HIV transmission [4], [7], [8]. This is of particular importance in sub-Saharan Africa where HIV prevalence rates are at their highest. Despite the roll-out of ART programmes in this region, ART coverage is still low [9]. Many HIV-infected patients seek healthcare with considerably more advanced immunodeficiency and may present with clinically different HHV-related diseases compared to individuals in resource-rich countries. In contrast to developed countries, where HHV seroprevalence and occurrence of associated diseases are well-documented, there is only scarce information available on the seroprevalence and risk factors of HHV infections in sub-Saharan Africa. This information is important to provide an indication of the burden of HHV infections which is particularly relevant for HIV-infected individuals who are known to be at increased risk for development of HHV-induced disease due to reactivation.

## Material and Methods

### Study setting and population

Study participants were recruited between September 2012 and January 2013 at primary healthcare (PHC) facilities across the Mopani District (Limpopo Province, South Africa), where the two main ethnic groups are Sotho (46%) and Shangaan (44%) [10]. Participating PHC facilities were selected by ratio of population-size of each of five sub-districts with a minimum of two PHC facilities/sub-district. Within each sub-district, PHC facilities were selected based on the number of patients on the ‘wellness, pre-ART programme’, geographic location and size of the catchment area. Individuals who had an indication to draw blood for determination of CD4 count (CD4 T-cells/mm^3^ blood) were eligible for this study. Criteria to participate were adult age (i.e. 18 years and older) and no prior ART exposure. Low educational status and low financial income were defined as having no education or primary school only, and persons with a government grant as the main source of income, respectively. The study was performed according to the tenets of the Helsinki Declaration, approved unconditionally by the Human Research Ethics Committee (Medical) of the University of the Witwatersrand, Johannesburg, South Africa (reference number: M120546), and written informed consent was obtained from all participants.

### Study and laboratory procedures

Demographic and clinical data were collected upon inclusion (Table 1). A whole blood sample was drawn and diagnostic CD4 counts were determined using the Cytomics FC 500 MPL platform (Beckman Coulter). Serological analysis was performed at the Laboratory of Viroscience at the Erasmus Medical Center in Rotterdam, The Netherlands. HHV-specific serum IgG titres were determined using Serion ELISA classic tests (Virion Serion) on the Anthos-Labtec AR8001 platform (Anthos Labtec). ELISAs were performed and data interpreted (i.e. seropositive, borderline or seronegative) per manufacturer's guidelines (Virion Serion).

**Table 1:** Demographic characteristics of study participants (n = 405).

Characteristic	
Gender (male)	72 (18)
Age in years (mean (SD))	38 (11)
Ethnicity	
Shangaan	255 (65)
Sotho	138 (35)
Marital status	
Never married	243 (60)
Married	129 (32)
Divorced or widowed	33 (8)
Has children	352 (87)
Individuals in household	5.1 (2.3)
Low educational status	192 (47)
Currently employed	103 (25)
Low financial income	184 (46)
In-house access to drinking water	25 (6.2)
In-house access to latrine	20 (5.0)
Keeps livestock	24 (5.9)
CD4 cell count in cells/mm^3^ (mean (SD))	382 (226)
Clinical HIV-stage^a^	
Stage 1	344 (85)
Stage 2	25 (6.0)
Stage 3	35 (9.0)
Stage 4	0 (0)

Data are presented as number (%) unless otherwise indicated. SD  =  standard deviation; HIV  =  human immunodeficiency virus.

a Clinical HIV-staging was done according to the WHO Clinical Staging of HIV/AIDS [11].

### Statistical analysis

Clinical and laboratory data were double-entered and validated using EPI-INFO version 3.5.4 (Centers for Disease Control). Description of study population and seroprevalence is done using number with proportion and mean with standard deviation. To identify factors associated with HHV-specific positive serostatus, excluding persons with borderline ELISA results, univariate analysis was performed using Chi-squared test, or Fisher's Exact test if appropriate, for categorical variables and Mann-Whitney and Student T-test for continuous variables. Data are presented as odds ratio (OR) with 95% confidence interval (CI), mean with standard deviation (SD) or as median. To identify factors independently associated with seropositive status variables with *p*-values <0.10 in univariate analysis, and age and gender as potential confounders, were included in multivariate analysis using logistic regression (forward Likelihood Ratio) with HHV seropositivity as dichotomous measure of outcome. The potential correlation of log2 HHV IgG titres with age and CD4 count was analysed using Spearman's correlation coefficient. Multiple linear regression analysis was performed to determine potential association of age and CD4 count, adjusting for gender and ethnicity, with the log2 HHV titre as continuous measure of outcome, providing assumptions for linear regression were met. Statistical analyses were conducted using PASW Statistics and *p*-values <0.05 were considered statistically significant.

## Results

### Study population

The study population of 405 HIV-infected and ART-naïve adults consisted of 72 (18%) men and 333 (82%) women (Table 1). The mean age was 37.9±11.5 years. Men were significantly older (mean age of 42 versus 37 years, *p* = 0.002), more often employed (age-adjusted OR (aOR) = 4.5; 95% CI: 2.6–7.7, *p*<0.001), were or had been married (aOR = 1.8; 95% CI: 1.0–3.1, *p*<0.05) and had lower mean CD4 counts than women (306 vs. 398 cells/mm^3^; *p*<0.001). Most participants had children (87%) and some had limited access to in-house drinking water (6.2%) and latrine (5.0%). Thirty (8%) participants were newly diagnosed for HIV infection, 354 (87%) attended the ‘wellness, pre-ART programme’ and 21 (5%) individuals attended the clinic for baseline results before ART initiation. Mean CD4 count was 382±226 CD4 T-cells/mm^3^ and fifty percent of participants had CD4 counts ≤350 cells/mm^3^, which is the threshold in South Africa to initiate ART [12].

### Seroprevalence of HHVs

HHV seroprevalence was determined in 402 samples (three were unavailable after transport); serum HHV ELISA data are summarized in Table 2. Excluding borderline results, HHV seroprevalence was very high: HSV-1 98% (95% CI: 96–99%); HSV-2 87% (95% CI: 83–90%); VZV 89% (95% CI: 86–92%) and 100% for both EBV and CMV. Seroprevalence was similar between individuals with CD4 count below and above 350 cells/mm^3^ for HSV-1 (98% vs. 97%; p = 0.33), HSV-2 (89% vs. 84%; p = 0.17) and VZV (91% vs. 88%; p = 0.35). Neither HHV seropositive status with CD4 count nor clinical HIV and HHV-related clinical symptoms was associated at time of sampling. The frequency of reported HHV-related clinical symptoms was low and not associated with the respective HHV seropositive status. History of chickenpox was reported by 72 (20%) of VZV seropositive and 5 (12%) of VZV seronegative individuals (*p* = 0.2) and herpes zoster by 50 (14%) and 4 (10%), respectively. Among HSV-1 seropositive individuals a history of cold sores and oral lesions was reported by 54 (15%) and 49 (14%) study participants, respectively. HSV-1 seronegative individuals did not report these diseases. Anogenital lesions were reported by 77 (24%) and 10 (20%) HSV-2 seropositive and seronegative study participants, respectively.

**Table 2:** Seroprevalence of human herpesviruses in HIV-infected individuals (n = 402).

	HSV-1	HSV-2	VZV	EBV	CMV
**Positive**	361 (90)	316 (79)	353 (88)	400 (99.5)	402 (100)
**Borderline**	32 (8.0)	37 (9)	7 (1.7)	2 (0.5)	0 (0)
**Negative**	9 (2.2)	49 (12)	42 (10)	0 (0)	0 (0)

Data are presented as number (%). HSV  =  human simplex virus; VZV  =  varicella zoster virus; EBV  =  Epstein-Barr virus; CMV  =  cytomegalovirus.

### Factors associated with positive HHV serostatus

Crude clinical and demographic factors associated with individuals' HHV seropositive vs. seronegative status for HSV-1, HSV-2 and VZV are summarized in Table 3. The 100% seroprevalence for EBV and CMV precluded calculation of such factors. Adjusting for age and gender, lack of in-house access to drinking water (aOR = 7.7; 95% CI: 1.8–33, *p* = 0.006) and Shangaan ethnicity (aOR = 2.4; 95% CI: 1.2–4.5, *p* = 0.008) were independently associated with positive HSV-1 and HSV-2 serostatus, respectively. Low educational status (aOR = 4.0; 95% CI: 1.8–9.0, *p* = 0.01), having children (aOR = 2.2; 95% CI: 1.0–4.9, *p* = 0.04) and a higher number of children in the household (*p* value = 0.007) were independently associated with VZV seropositivity, including adjustment for age and gender (data not shown).

**Table 3:** Crude risk factors associated with HSV-1, HSV-2, and VZV serostatus.

	HSV-1 serology				HSV-2 serology				VZV serology			
	Positive (n = 361)	Negative (n = 9)	Crude odds ratio (95% CI)	p-Value	Positive (n = 316)	Negative (n = 49)	Crude odds ratio (95% CI)	p-Value	Positive (n = 353)	Negative (n = 42)	Crude odds ratio (95% CI)	p-Value
Age in years	38 (11)	34 (9)	na	0.4	39 (12)	34 (10)	na	0.02	39 (11)	32 (10)	na	0.001
Gender												
Men	62 (98)	1 (2)	1.7 (0.2–13.5)	1.0	53 (86)	9 (14)	0.9 (0.4–2.0)	0.8	61 (86)	10 (14)	0.7 (0.3–1.4)	0.3
Women	299 (97)	8 (3)	1		262 (87)	40 (13)	1		292 (90)	32 (10)	1	
Ethnicity												
Shangaan	235 (98)	6 (2)	0.7 (0.1–3.4)	1.0	206 (91)	20 (9)	2.4 (1.3–4.5)	0.006	222 (89)	27(11)	0.8 (0.4–1.7)	0.6
Sotho	116 (98)	2 (2)	1		103 (81)	24 (19)	1		122 (91)	12 (9)	1	
Low educational status	166 (46)	1 (11)	6.8 (0.8–55)	0.07	148 (47)	22 (45)	1.1 (0.6–2.0)	0.8	178 (50)	8 (19)	4.3 (1.9–9.6)	<0.0001
Currently employed	91 (25)	2 (11)	1.2 (0.2–4.8)	1.0	81 (26)	13 (27)	1.0 (0.5–1.9)	0.9	90 (26)	11 (26)	1.0 (0.5–2.0)	0.9
Low financial income	163 (45)	1 (11)	6.7 (0.8–54)	0.08	145 (46)	17 (35)	1.6 (0.9–3.0)	0.1	163 (46)	16 (38)	1.4 (0.7–2.7)	0.3
Marital status												
Never married	220 (98)	5 (2)	1.2 (0.3–4.7)	0.7	191 (88)	27 (12)	1.2 (0.7–2.3)	0.5	206 (87)	32 (13)	0.4 (0.2–0.9)	0.03
Married, widowed or divorced	141 (97)	4 (3)	1		125 (85)	22 (15)	1		147 (94)	10 (6)	1	
In-house access to drinking water	22 (6.1)	3 (33)	0.13 (0.03–0.55)	0.02	17 (5.4)	7 (14)	0.3 (0.1–0.9)	0.03	23 (6.5)	2 (4.8)	1.4 (0.3–6.1)	1.0
In-house access to latrine	17 (4.7)	2 (22)	0.17 (0.03–0.90)	0.07	13 (4.1)	6 (12)	0.3 (0.1–0.9)	0.03	17 (4.8)	3 (7.1)	0.7 (0.2–2.3)	0.5
Clinical HIV-stage												
Stage 1	304 (97)	9 (3)	-	0.4	265 (86)	42 (14)	0.7 (0.3–1.8)	0.5	302 (90)	34 (10)	1.2 (0.5–2.9)	0.7
Stages 2-4	56 (100)	0 (0)	1		51 (89)	6 (11)	1		51 (88)	7 (12)	1	
CD4 cell count in cells/mm3	373 (219)	491 (285)	na	0.1	381 (236)	398 (188)	na	0.6	386 (228)	374 (224)	na	0.8
History of cold sores	54 (15)	0 (0.0)	0.97 (0.95–0.99)	0.4	-	-	-	-	-	-	-	-
History of oral lesions	49 (14)	0 (0.0)	0.97 (0.95–0.99)	0.6	44 (14)	10 (20)	0.6 (0.3–1.4)	0.2	-	-	-	-
History of anogenital lesions	87 (24)	1 (11)	2.5 (0.3–21)	0.7	77 (24)	10 (20)	1.3 (0.6–2.6)	0.5	-	-	-	-
History of chickenpox	-	-	-	-	-	-	-	-	72 (20)	5 (12)	1.9 (0.7–5.0)	0.2
History of vesicular rash	-	-	-	-	-	-	-	-	50 (14)	4 (10)	1.6 (0.5–4.6)	0.4

Data are shown as numbers (%) or mean (sd). Crude odds ratios were calculated for demographic and clinical characteristics between positive and negative serological status. CI, Confidence interval; *p*-Value, Pearson Chi-square; na, not applicable; VZV, varicella zoster virus; HSV-1, herpes simplex virus 1; HSV-2, herpes simplex virus 2.

**Figure 1:**
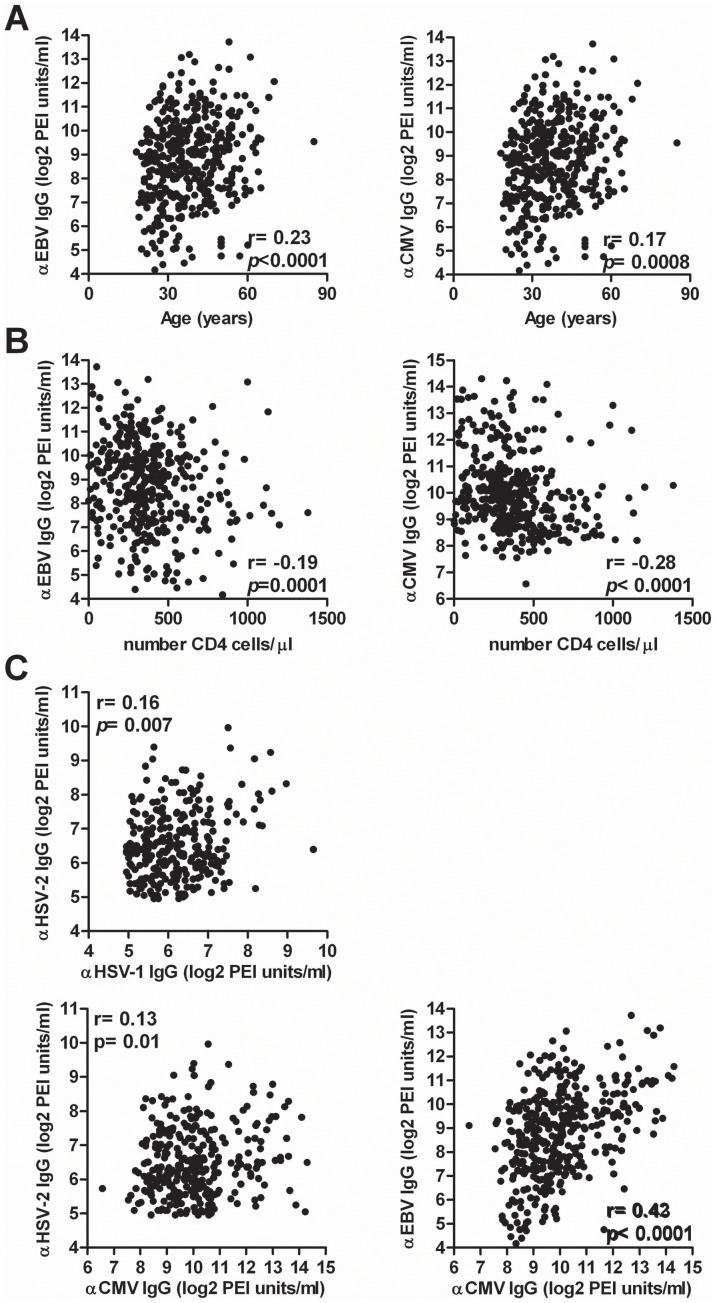
Scatter plots of factors associated with human herpesvirus-specific serum IgG titres. Correlation between serum EBV and CMV IgG titres and the individual's (**A**) Age and (**B**) CD4 cell count. (**C**) Correlation between serum IgG titres of different human herpesviruses. Serum IgG titres, presented as binary logarithmic PEI/ml values, were calculated based on corresponding reference sera from the Paul-Ehrlich Institute (Erlangen, Germany). The Spearman correlation test was used for statistical analysis. HSV-1, herpes simplex virus 1; HSV-2, herpes simplex virus; VZV, varicella zoster virus; EBV, Epstein-Barr virus and CMV, cytomegalovirus.

Next, we assayed potential correlations between HHV-specific IgG titres and paired individual's laboratory and clinical data (Fig. 1). EBV and CMV IgG titres correlated significantly with age and CD4 count. Increasing age was associated with higher EBV (*R* ^2^ = 0.05, *p*<0.001) and CMV IgG titres (*R* ^2^ = 0.03, *p* = 0.0008) (Fig. 1A), whereas increasing CD4 count was negatively associated with EBV (*R* ^2^ = −0.04, *p* = 0.0001) and CMV IgG titres (*R* ^2^ = 0.08, *p*<0.0001) (Fig 1B). There was no association of CD4 count with HSV-1, HSV-2 and VZV IgG titre (see Fig. S1). Higher HSV-1 IgG titres were detected among men (median 88 U/mL versus 66 U/mL in women; *p* = 0.001). Furthermore, HSV-1 and HSV-2 (*R* ^2^ = 0.03, *p* = 0.007), CMV and VZV (*R* ^2^ = 0.02, *p* = 0.013) and CMV and EBV (*R* ^2^ = 0.18, *p*<0.0001) IgG titres were positively correlated (Fig. 1C). No associations of age, gender and ethnicity were observed between IgG titres for the other HHVs.

Finally, multivariate analysis of determinants of HHV titre was performed for age, gender, ethnicity and CD4 count. In individuals with similar gender and ethnicity, there was an association for every one year increase in age (0.17 unit) and every cell reduction in CD4 count (−0.25 unit) with CMV IgG titre. Also, for every one year increase in age the EBV IgG titre was associated with a 0.17 unit increase in titre in subjects comparable in gender, ethnicity and CD4 count, whereas male gender was associated with a decrease in HSV-1 IgG tire (−0.17 unit; see Table S1). No associations between HSV-2 and VZV IgG titres and the aforementioned factors were observed.

## Discussion

This study reports on the seroprevalence of HSV-1, HSV-2, VZV, EBV and CMV among HIV-infected and ART-naïve adults in rural South Africa. We showed that HHV seroprevalence in this population is very high. We identified several demographic factors that were associated with a seropositive status for HSV-1, HSV-2 and VZV, but did not observe that clinical history of HHV infection is predictive for the individual's HHV serostatus.

We observed a relatively high seroprevalence of HHVs which confirms observations in other studies addressing seroprevalence of these viruses among HIV-infected individuals in Africa. Estimates of HSV-1 and HSV-2 seroprevalence among adult HIV-infected individuals vary across Africa, ranging from 65 to 90% in studies from South Africa, Kenya, Lesotho and Tanzania [13]–[17]. High prevalence rates (>85%) have also been reported for VZV, CMV and EBV in HIV-infected adult individuals from several African countries [13], [14], [18]. These infections may be acquired in early childhood as illustrated by the 100% CMV seroprevalence among a small cohort of city-dwelling HIV-infected children in Kenya [19]. As such, a generally high seroprevalence of HHVs is manifest among adults with HIV-infection across Africa. It should be noted that we recruited a selected group of individuals in our study (HIV-infected and ART-naïve adults) and that our findings should not be extrapolated to the general adult African population including either HIV negative or HIV positive adults on ART therapy. Since HIV-infection is associated with increased HHV seroprevalence, it may be expected that seroprevalence of these viruses in the general population would be lower as for example shown for HSV and EBV [1], [20].

We identified several factors associated with seropositive status for 5 individual HHVs, but these should be interpreted with caution as seroprevalence was very high, resulting in a relatively small group of seronegative participants, and the effect of chance associated with multiple comparison cannot be ruled out. In addition, we like to point out that the use of prevalence odds ratio instead of prevalence ratio may have resulted in an overestimation of the observed associations; although there are pros and cons to both measures of effect [21], [22]. Low socioeconomic status - represented here as lack of in-house access to drinking water - was appeared to be associated with HSV-1 seropositivity. Shangaan ethnicity was independently associated with HSV-2 seropositivity, suggesting a higher HSV-2 infection rate among Shangaan compared to Sotho. This potential association may be due to different sexual risk behaviour between these ethnic groups [unpublished data]. However, our study did not confirm previously described associations of seropositivity for HSV-2 and female gender, age, and other socioeconomic factors, but this may be due to the high seroprevalence in our study population [23]–[27]. As reported earlier, VZV seropositive status associated independently with age, low educational status, having children and number of children, and number of individuals in the household [28]–[32]. Notably, no association was observed between the individual's seropositive HHV status and a history of corresponding HHV-specific clinical symptoms. This could be due to recall bias as some infections would have occurred in early childhood (e.g. varicella); other infections can have a subclinical course (e.g. CMV, HSV) without ever presenting with clear symptoms or being recognised by the individual. As such, based on the poor predictive value, it would not be possible to identify individuals with seropositive status, and thus at increased risk of HHV reactivation and complications, based on clinical history taking.

We observed a moderate positive associations between EBV and CMV IgG titres and age. As most individuals are infected with HHV during childhood, increasing reactivation rates of the respective HHVs during ageing and/or progress of HIV infection may have attributed to this association [33], [34]. Indeed, age-related waning of cellular immunity and higher reactivation rates of latent HHVs resulting in increased HHV-specific IgG and IgM titres have been reported [33], [35], [36]. The negative association of EBV and CMV IgG titres with CD4 count is in line with two recent studies describing significant increase of activated HHV-specific CD4 T-cells in HIV-infected individuals compared to HIV naive individuals [37], [38]. Decreasing CD4 count due to HIV replication is associated with activation of CMV- and EBV-specific CD4 T-cells as part of chronic immune activation, which may subsequently stimulate humoral responses and produce higher IgG titres in time.

The observed weak, though significant associations between serum IgG levels of different HHVs suggest that risk factors of reactivation leading to higher IgG titres partially overlap. The associations between HSV-1 and HSV-2, and EBV and CMV IgG titres may in part be attributed to the anatomic and cellular site and corresponding immune responses involved in controlling viral latency. Both HSV types establish latency in sensory neurons, whereas lymphocytes are the main cell type of CMV (hematopoietic progenitor cells) and EBV (B-cells) latency [39]–[41]. HIV infection may inhibit specific immune cell types and mechanisms that are commonly involved in controlling either latent neurotropic (HSV-1 and HSV-2) and lymphotropic (EBV and CMV) herpesviruses. Moreover, the positive association between age and CD4 count with both EBV and CMV strengthen this assumption that immunity to both viruses is entangled, which warrants further studies [36]. However, contrary to our results, the observed association in the latter study was between CMV and HSV-1 IgG titres and was only significant in individuals <45 years old. In addition, CMV seropositivity, but not CMV IgG titres, has been associated with increased VZV IgG titres [34]. Since all individuals in our study cohort were CMV seropositive a similar association analysis could not be performed.

There are several limitations to this study. First, PHC facilities were not randomly selected within the five sub-districts in Mopani, which may have resulted in some degree of bias in obtaining a population estimate for the Mopani District. However, since PHC facilities were sampled by ratio of sub-district's population-size with a minimum of two PHC facilities per sub-district and no geographical variation was observed, we consider our study cohort representative for HIV-infected individuals attending PHC facilities in the Mopani district. Second, only individuals attending PHC facilities were included, a population that may be different for some demographic characteristics compared to those not seeking healthcare. Generally, the latter group ultimately presents with lower CD4 counts and higher risk of HHV-related disease suggesting an under- rather than overestimate of seroprevalence. Third, considerably more women than men were included in this study, but statistical analyses were adjusted for gender. Fourth, the use of self-reported clinical history of HHV infection may have resulted in some degree of recall bias, especially since all participants were adults. Finally, we did not include HHVs type 6 and 8 in our seroprevalence study; HHV-8 in particular is associated with morbidity (Kaposi sarcoma) in HIV-infected individuals.

HHV infections have currently limited priority and awareness in the (pre-)ART programme of South Africa. The herein reported high HHV seroprevalence and consequently high risk for HHV-related diseases among HIV-infected individuals warrant increased awareness among healthcare workers in rural South Africa for early clinical signs of these conditions to initiate prompt antiviral treatment: e.g. early diagnosis and treatment of herpes zoster ophthalmicus to prevent corneal blindness [4], [6]. In conclusion, seroprevalence of HHVs in rural South Africa is very high and recognition and awareness of HHV-related diseases is warranted.

## Supporting Information

Figure S1 **Scatter plots of age and CD4 cell count with specific serum IgG titres for HSV-1, HSV-2 and VZV.** Serum IgG titres, presented as binary logarithmic PEI/ml values, were calculated based on corresponding reference sera from the Paul-Ehrlich Institute (Erlangen, Germany). The Spearman correlation test was used for statistical analysis. HSV-1, herpes simplex virus 1; HSV-2, herpes simplex virus; VZV, varicella zoster virus.
(TIF)

Table S1 **Results of multivariate linear regression analysis of age, gender, ethnicity and CD4 cell count with log2 IgG titre of individual human herpes viruses.**
(DOCX)
