# Supplementary figures and images for: High Seroprevalence of Human Herpesviruses in HIV-Infected Individuals Attending Primary Healthcare Facilities in Rural South Africa
Source: PLoS One. 2014 Jun 10;9(6):e99243. doi: 10.1371/journal.pone.0099243 (PMC4051661; doi:10.1371/journal.pone.0099243)

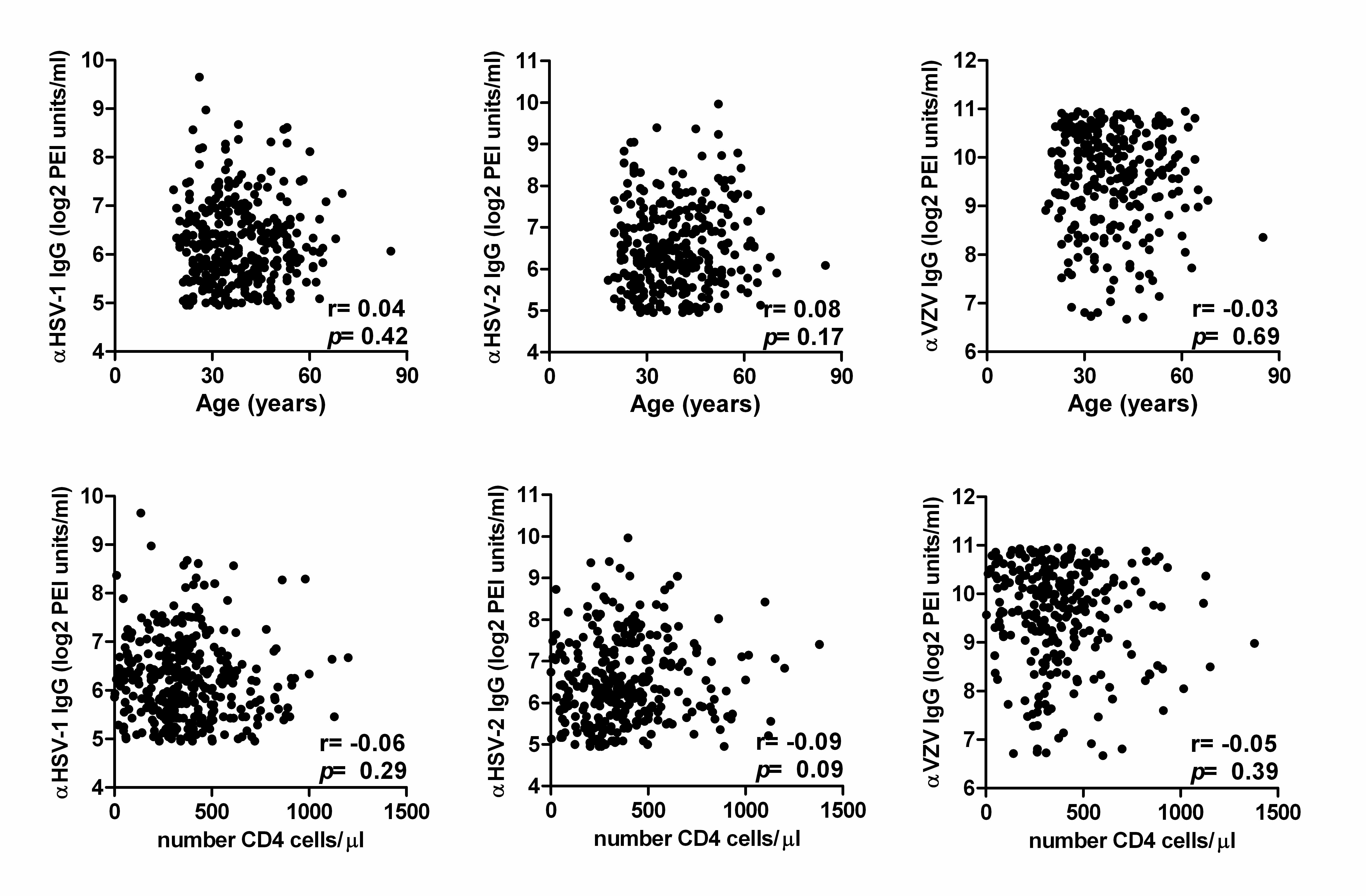

Supplement: Figure S1 — Scatter plots of age and CD4 cell count with specific serum IgG titres for HSV-1, HSV-2 and VZV. Serum IgG titres, presented as binary logarithmic PEI/ml values, were calculated based on corresponding reference sera from the Paul-Ehrlich Institute (Erlangen, Germany). The Spearman correlation test was used for statistical analysis. HSV-1, herpes simplex virus 1; HSV-2, herpes simplex virus; VZV, varicella zoster virus. (TIF) [file pone.0099243.s001.tif]
