# Supplementary material for: High Seroprevalence of Human Herpesviruses in HIV-Infected Individuals Attending Primary Healthcare Facilities in Rural South Africa
Source: PLoS One. 2014 Jun 10;9(6):e99243. doi: 10.1371/journal.pone.0099243 (PMC4051661; doi:10.1371/journal.pone.0099243)
Supplement: Table S1 — Results of multivariate linear regression analysis of age, gender, ethnicity and CD4 cell count with log2 IgG titre of individual human herpes viruses. (DOCX) [file pone.0099243.s002.docx]

**Table S1. Results of multivariate linear regression analysis of age, gender, ethnicity and CD4 cell count with log2 IgG titre of individual human herpes viruses**

|  | | **Beta** | **P-value** | **R^2^** | **F-statistic** | **P-value** |
| --- | --- | --- | --- | --- | --- | --- |
| **HSV-1 IgG titre** | |  |  |  |  |  |
|  | ***Intercept*** | 6.96 |  |  |  |  |
|  | **Age** | -0.02 | 0.8 | 0.035 | 3.13 | 0.02 |
|  | **Gender** | -0.17 | 0.002 |  |  |  |
|  | **Ethnicity** | -0.01 | 0.9 |  |  |  |
|  | **CD4 cell count** | -0.04 | 0.5 |  |  |  |
|  | |  |  |  |  |  |
| **HSV-2 IgG titre** | |  |  |  |  |  |
|  | ***Intercept*** | 6.09 |  |  |  |  |
|  | **Age** | 0.09 | 0.1 | 0.014 | 1.11 | 0.4 |
|  | **Gender** | 0.04 | 0.5 |  |  |  |
|  | **Ethnicity** | -0.06 | 0.3 |  |  |  |
|  | **CD4 cell count** | 0.06 | 0.3 |  |  |  |
|  | |  |  |  |  |  |
| **VZV-1 IgG titre** | |  |  |  |  |  |
|  | ***Intercept*** | 10.6 |  |  |  |  |
|  | **Age** | -0.05 | 0.4 | 0.006 | 0.54 | 0.7 |
|  | **Gender** | -0.05 | 0.3 |  |  |  |
|  | **Ethnicity** | -0.02 | 0.38 |  |  |  |
|  | **CD4 cell count** | -0.03 | 0.6 |  |  |  |
|  | |  |  |  |  |  |
| **EBV IgG titre** | |  |  |  |  |  |
|  | ***Intercept*** | 7.31 |  |  |  |  |
|  | **Age** | 0.17 | <0.001 | 0.027 | 3.66 | 0.006 |
|  | **Gender** | -0.03 | 0.5 |  |  |  |
|  | **Ethnicity** | -0.04 | 0.5 |  |  |  |
|  | **CD4 cell count** | -0.07 | 0.2 |  |  |  |
|  | |  |  |  |  |  |
| **CMV IgG titre** | |  |  |  |  |  |
|  | ***Intercept*** | 9.34 |  |  |  |  |
|  | **Age** | 0.17 | 0.001 | 0.076 | 8.92 | <0.001 |
|  | **Gender** | 0.04 | 0.4 |  |  |  |
|  | **Ethnicity** | 0.03 | 0.6 |  |  |  |
|  | **CD4 cell count** | -0.25 | <0.001 |  |  |  |

**Note.** HSV, herpes simplex virus; VZV, varicella-zoster virus; CMV, cytomegalovirus; EBV, Epstein-Barr virus.
